# Serum Anti-Mullerian hormone and embryo morphokinetics detecting by time-lapse imaging: A comparison between the polycystic ovarian syndrome and tubal factor infertility

**Published:** 2018-08

**Authors:** Nasim Tabibnejad, Mehrdad Soleimani, Abbas Aflatoonian

**Affiliations:** *Research and Clinical Center for Infertility, Yazd Reproductive Sciences Institute, Shahid Sadoughi University of Medical Sciences, Yazd, Iran.*

**Keywords:** Anti-Mullerian hormone, Embryo morphokinetic, Pregnancy outcome, Time-lapse, PCOS

## Abstract

**Background:**

Anti-Mullerian hormone (AMH) is considered as a good marker for quantitative evaluation of ovarian response to the stimulation during assisted reproductive technology cycles.

**Objective:**

To evaluate the association between serum AMH level and embryo morphokinetics using time-lapse imaging and intracytoplasmic sperm injection (ICSI) outcomes in women with polycystic ovarian syndrome (PCOS).

**Materials and Methods:**

We evaluated a total of 547 embryos from 100 women underwent ICSI cycles; 50 women with PCOS and 50 women with tubal factor infertility. Serum AMH level was measured in all participants. Time-laps records were annotated for time to pronuclear fading (tPNf), time to 2-8 cells (t2-t8), reverse cleavage, direct cleavage, and also for the presence of multinucleation.

**Results:**

AMH was negatively correlated with t5, t8, and the third cell cycle (p=0.02, p=0.02, and p=0.01; respectively) in PCOS group. AMH had no correlation with embryo kinetics in infertile women with tubal factor infertility. Moreover, AMH level is similar between embryos with and without direct cleavage as well as reverse cleavage and Multinucleation in both groups. The Receiver operating characteristic curves analyses indicated that AMH was not an accurate predictor of clinical pregnancy as well as a live birth (AUC=0.59 [95% CI, 0.42-0.76]) in PCOS women. However, in the women with tubal factor infertility AMH showed a fair prediction value for clinical pregnancy (AUC=0.64 [95% CI, 0.48-0.82]) along with the live birth (AUC=0.70 [95% CI, 0.55-0.85]).

**Conclusion:**

Some of the time-lapse embryo parameters may be related to the AMH concentration. However, AMH is not an accurate tool to predict the ICSI outcomes in PCOS women.

## Introduction

Anti-Mullerian hormone (AMH); a part of the transforming growth factor-beta superfamily structured as a dimeric glycoprotein (1). AMH was secreted by granulosa cells from the pre-antral and antral follicle and plays a key role in the regulation of follicular development (2). The high sensitivity of AMH for prediction of ovarian reserve is well established (3-5). Also, it is considered as a good marker for quantitative evaluation of ovarian response to the stimulation during assisted reproductive technology (ART) cycles (1, 6). However, the association of AMH with oocyte and embryo quality as well as ART outcome is still controversial. Several reports found a positive correlation between AMH level and oocyte/embryo quality (6-8) nevertheless, the other studies did not indicate such an association (9-11). In addition, the correlation between AMH and implantation, pregnancy, and live birth after assisted reproduction has been shown in some previous reports (12, 13), however, this relationship did not confirm by others (8, 14). 

On the other hand, women with polycystic ovary syndrome (PCOS) presented a remarkable increased number of developing follicles compared with normal women (15). Moreover, these patients showed a circulating AMH level two to three times higher than normal women (16). A recent meta-analysis reported a poor ability of AMH in the prediction of clinical pregnancy in PCOS patients (17). It should be considered that all of the above-mentioned studies assessed embryo quality using standard morphological criteria. However innovative and non-invasive techniques for embryo evaluation such as time-lapse imaging provide additional details regarding embryo morphokinetics compared to the traditional embryo assessment (18, 19).

The aim of this study was to evaluate the association between serum AMH level and embryo morphokinetics using time-lapse monitoring along with ART outcomes in PCOS women.

## Materials and methods

**Participants**

We prospectively evaluated 100 sequential cycles of intra-cytoplasmic sperm injection (ICSI) in which embryo development was monitored by a time-lapse embryoscope. Enrolled women were 50 PCOS patients and 50 women with tubal factor (TF) infertility. The patients were included the study between April 2016 and April 2017. Women with the age less than 43 yr; who had fewer than three previous failed in vitro fertilization (IVF)/ICSI cycles and scheduled for day 3 embryo transfer were included the study. The women had at least one zygote (2-pronuclear [2PN]) obtainable on day 1 for time-lapse imaging.

PCOS women were diagnosed based on the Rotterdam criteria (20). TF infertility was confirmed by hysterosalpingogram or laparoscopy in women who had removed fallopian tube(s) due to tubal pregnancy and proximal tubal adhesions. Endometriosis and severe male factor (total motile sperm <1 million) were proposed as exclusion criteria.

**AMH measurement**

A venous blood sample was collected from all participants and basal AMH was measured by a commercial ELISA kit (AMH/MSI ELISA; AnshLabs, TX, USA). One quantity of each calibrator, control, or test samples was regulated respectively with four parts of AMH/MSI assay buffer, and no dilution factor was applied. Based on the kit instruction, we diluted any sample that reads greater than the maximum calibrator with sample diluent. 

**Ovarian stimulation**

The majority of women (90%) were stimulated using an antagonist protocol (21), and the others administered by an agonist protocol (3%) (22) or a microdose flare protocol (7%) (23). Follicular growth was monitored by transvaginal ultrasound. An intramuscular injection of 10,000 IU of human chorionic gonadotropin (hCG) (Pregnyl®, Organon, Oss, Netherlands) was ordered when at least three follicles reached a diameter of ≥18 mm. Oocyte retrieval was performed by the ultrasound guide 36 hr after the hCG administration. 

**Laboratory procedure**

At the time of pickup, oocytes were incubated in culture medium (G-IVF; VitroLife, Kungsbacka, Sweden) concealed with mineral oil (Ovoil; VitroLife) at 37^o^C, with 6% CO_2_ for 2-3 hr. 80 IU/ml hyaluronidase (Sigma Co, USA) was applied to assistance denudation of cumulus cells. The husband’s prepared sperm was used for injection of Mature (MII) oocytes. The injected oocytes cultured in a standard incubator at 37^o^C with 6% CO_2_ in fresh droplets of G1 (Vitrolife Co., Sweden) and covered with mineral oil (Reploline Co., Germany) overnight.

A nine-well embryo culture dish (Primo Vision dish, VitroLife, Sweden) was provided with 40 µl G1+ medium, and shielded with 3 ml of mineral oil and adapted overnight for the culture of the fertilized eggs on the next day. Fertilization was evaluated by detection of the two pronuclei (2PN) and two polar bodies 16-18 hr after insemination. Typically fertilized zygotes were transferred to the prepared Primo Vision dish for culture inside the Embryoscope (Primo Vision, VitroLife, Sweden). The culture dish was placed in a time-lapse microscope at 37^o^C, 5% O_2_ and 6% CO_2_ for the following 3 days without media alteration or refreshment.

**Time-lapse Imaging system**

For each embryo, several images were attained every 10 min in seven focal planes. The accurate timings from the point of ICSI were measured by Primo Vision Embryo Viewer Software: time to pronuclear fading (tPNf), time to 2 cells (t2), 3 cells (t3), 4 cells (t4), 5 cells (t5), 6 cells (t6), 7 cells (t7) and 8 cells (t8). Supplementary kinetic factors were calculated: duration of the second cell cycle (cc2=t3-t2), third cell cycle (cc3=t5-t3) and time to inclusive first, second and third synchronous divisions, s1 (t2- tPNf), s2 (t4-t3) and s3 (t8-t5). Multinucleation (MN); (presence of more than one nucleus in a blastomere) was annotated. In addition, two detected cleavage anomalies were direct cleavage (DC); when a single blastomere divided directly from 1-3 cells in earlier than 5 hr and reverse cleavage (RC); when a blastomere was reabsorbed after cleavage. 

**Embryo selection and transfer**

Embryos were selected for transfer on day 3 according to the morphologic criteria (the best morphology) and high marks delivered by the time-lapse records. Two embryos were transferred using an embryo transfer Labotect catheter (Labor-Technik-Göttingen GmbH, Gottingen, Germany). In the cases of bad quality embryos or patients' request up to three embryos were transferred (24). Other good quality embryos that were not assigned for transfer were cryopreserved. Furthermore, progesterone suppositories; (Cyclogest®) (Cox Pharmaceuticals, Barnstaple, UK) were ordered vaginally, 400 mg twice a day from the day of oocyte pick up until the detection of fetal heart activity by ultrasound in the 8^th^ wk of pregnancy. 

**Outcome measurement**

The primary outcome was the association between serum AMH level and embryo morphokinetics detecting by time-lapse embryoscope. The secondary outcome was the predictive value of AMH in the estimation of ICSI outcome. Chemical pregnancy was defined by positive β-hCG 14 days after embryo transfer and clinical pregnancy was approved by the observation of fetal heart activity by transvaginal ultrasonography 2-3 wk after positive β-hCG. Live birth was considered as a baby born alive living more than 30 days.

**Ethical consideration**

The study proposal was approved by the Ethics Committee of Yazd Reproductive Sciences Institute, Shahid Sadoughi University of Medical Sciences, Yazd, Iran (IR.SSU.RSI.REC.1396.26). The study was directed based on the Helsinki Declaration. A written informed consent form was signed by all the participants.

**Statistical analysis**

Quantitative variables were presented as mean±standard deviation (SD) and matched with Student's *t*-test and Mann-Whitney U-test according to their distribution pattern measured by the Kolmogorov-Smirnoff test. Qualitative variables were offered as percentages and compared by Chi-square test. A Spearman's rank correlation was computed to assess the relationships between AMH concentration and early cleavage timing in the developing embryos.

The Receiver operating characteristic curves (ROC) were applied to measure the predictive accuracy of AMH for clinical pregnancy and live birth. The model discriminative presentation was calculated by the area under the curve (AUC) of the ROC curve. 95% confidence intervals (CI) were calculated. All Analyses were performed using the Statistical Package for the Social Science version 20 for Windows (SPSS Inc, Chicago. IL, USA) and a p<0.05 was set to be significant.

## Results

289 embryos in the PCOS group and 258 embryos in the TF infertility group were collected from 100 ICSI treatment cycles. Participants' characteristics and cycle details are listed in table I. There were no significant differences between the two groups in the basic variables. The serum AMH level, number of retrieved, and MII oocytes were significantly higher in the PCO women compared to the TF group. The mean timings of tPNf, t2 to t8, along with cc2, cc3, and s1 to s3 was prolonged in the PCOS group compared to the women with TF infertility. The differences were statistically significant apart from cc2, cc3, s1 and s2 sets. Also, the incidence of morphological events including MN, RC, and DC was comparable between the two groups (data are not shown).

Correlation between AMH and embryo kinetic events was calculated to evaluate the relationship between AMH and embryo development. As expected, AMH was negatively correlated with age and positively correlated with the number of retrieved oocytes, number of matured oocytes and embryos in the two groups. With regards to the embryo kinetic timing, AMH was negatively correlated with t5, t8, and cc3 (p=0.02, p=0.02, and p=0.01; respectively) among PCOS women. AMH had no correlation with embryo kinetics in infertile women with TF infertility (Table II). 

**Figure 1:**
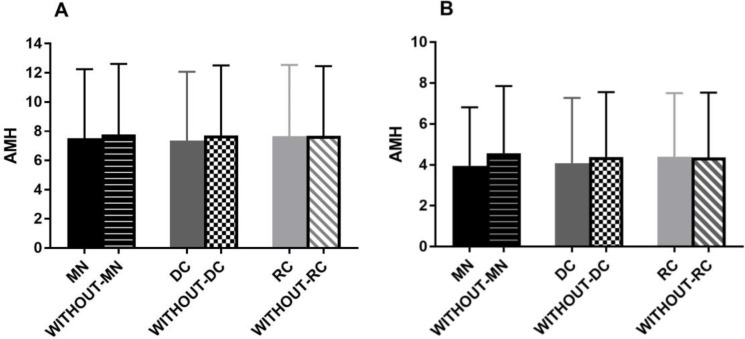
AMH concentration regarding embryo morphology events. AMH levels in embryos with MN and without MN, with DC and without DC and with RC and without RC in PCOS patients (A) as well as in TF patients (B). There is not any significant difference between the above analysis.

**Figure 2:**
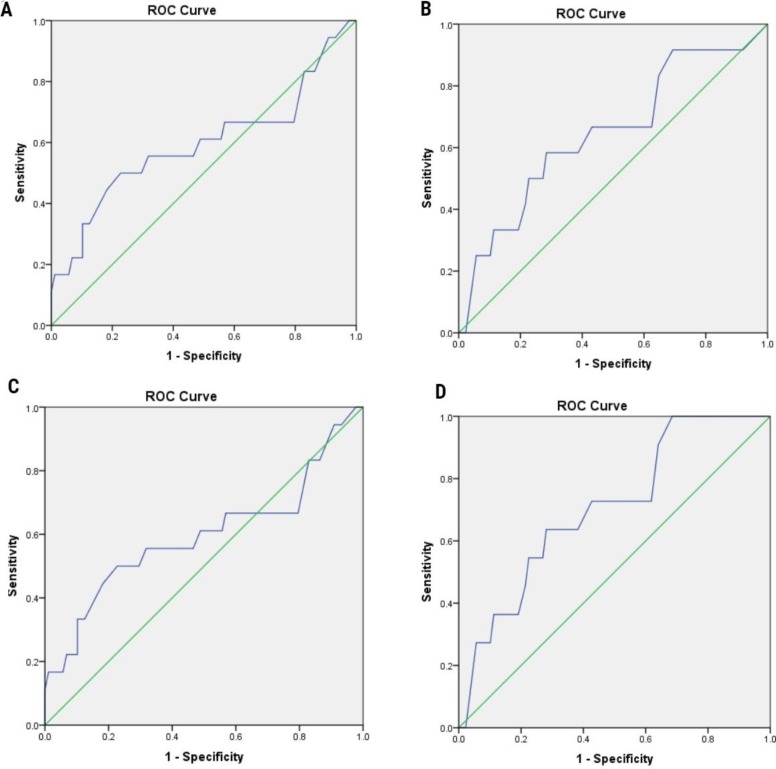
ROC curve analysis showing the predictive value of AMH for estimation of clinical pregnancy in the PCO patients (A); and tubal factor infertility (B). ROC curve analysis showing the predictive value of AMH for estimation of live birth in the PCOS women (C) as well as tubal factor infertility patients (D). The diagonal line is the reference line of no discrimination (AUC= 0.5

AMH was also investigated regarding embryo morphological abnormalities. The results showed that AMH level is similar between embryos with and without DC as well as RC and MN in both groups (Figure 1). Regarding the predictive value of AMH in the subject of the reproductive outcome, the ROC analyses indicated that AMH was not an accurate predictor of clinical pregnancy as well as a live birth (AUC=0.59 [95% CI, 0.42-0.76]) in PCOS women. However, in the women with TF infertility AMH showed a fair prediction value for clinical pregnancy (AUC=0.64 [95% CI, 0.48-0.82]) along with living birth (AUC=0.70 [95% CI, 0.55-0.85]) (Figure 2).

**Table I:** Patients characteristics and cycle specifics in two study groups

**Variable**	**PCOS group (n= 50)**	**Tubal factor group (n= 50)**	**p-value**
Age (yr)	30.04 ± 4.59	31.40 ± 4.99	0.524*
Length of infertility (yr)	6.92 ± 4.69	7.54 ± 4.67	0.416**
BMI (kg/m^2^)	26.06 ± 4.03	25.55 ± 3.73	0.435*
AMH (ng/ml)	7.67 ± 4.78	4.36 ± 3.16	0.000**
E_2_ (pg/ml)	2400.72 ± 1662.35	2101.90 ± 1615.09	0.215**
Oocytes retrieved	13.12 ± 7.80	9.46 ± 5.19	0.0098**
Mature MII oocytes	10.82 ± 6.95	8.06 ± 4.77	0.034**
Mean embryo transferred	2.16 ± 0.61	2.00 ± 0.57	0.256**

Data are presented as mean±SD.

PCOS= Poly cystic ovarian syndrome

BMI= Body mass index

AMH= Antimullerian hormone

E_2_= Estradiol

MII= Metaphase II

* PCOS vs. tubal factor group using Student’s t-test.

** PCOS vs. tubal factor group using Mann-Whitney U test.

**Table II:** Spearman's rank correlation analysis between AMH and number of oocytes, and embryos in addition to embryos' kinetics in two groups

**Variables**	**AMH**			
	**PCOS group (n= 289)**		**Tubal factor group (n= 258)**	
	**rs**	**p-values**	**rs**	**p-values**
Age (yr)	-0.69	0.000*	-0.49	0.000*
Oocytes retrieved	0.45	0.001*	0.40	0.003*
Mature MII oocytes	0.42	0.002*	0.35	0.011*
Number of embryos	0.40	0.004*	0.25	0.040*
Kinetic marker(hours post ICSI), tPNf	0.06	0.278	-0.07	0.234
t2	0.02	0.622	0.02	0.723
t3	0.04	0.485	0.10	0.098
t4	0.01	0.745	0.68	0.279
t5	-0.13	0.025*	0.02	0.872
t6	-0.08	0.143	0.09	0.144
t7	-0.05	0.375	0.05	0.392
t8	-0.13	0.024*	-0.03	0.623
cc2	0.02	0.696	0.10	0.108
cc3	-0.14	0.013*	-0.10	0.098
s1	-0.03	0.511	0.07	0.241
s2	-0.02	0.626	-0.09	0.147
s3	0.00	0.879	-0.06	0.284

AMH= Antimullerian hormone

PCOS= Poly cystic ovarian syndrome

rs= Correlation coefficient

ICSI: Intracytoplasmic sperm injection

tPNf= Time to pronuclear fading

t2= Time to 2 cells

t3= Time to 3 cells

t4= Time to 4 cells

t5= Time to 5 cells

t6= Time to 6 cells

t7= Time to 7 cells

t8= Time to 8 cells

cc2= Duration of the second cell cycle (t3-t2)

cc3= Duration of the third cell cycle (t5-t3)

s1, s2 and s3= Complete first, second and third synchronous divisions: s1: (t2-tPNf), s2 (t4-t3), and s3 (t8-t5)

* p<0.05.

AMH: Antimullerian hormone

MN: Multinucleation

DC: Direct cleavage

RC: Reverse cleavage

## Discussion

This is the first study that evaluates the correlation between serum AMH level and both early embryo morphokinetics and ICSI outcome in PCOS women. The results showed that embryo morphokinetic markers do not reflect AMH concentration and also revealed that AMH could not be used as a predictor for pregnancy and live birth in PCOS women. Up to now, there have been a number of publications considering the possible use of embryo morphokinetics to develop ART outcome (25, 26). 

Furthermore, several reports investigate the relationship between serum AMH and both oocyte and embryo quality and also pregnancy outcome in ART cycles (7, 8, 27). But only one recent study evaluated the correlation between AMH and embryo development using time-lapse parameters. Bhide and colleagues categorized all studied women according to the embryo morphokinetics into five groups. The authors could not find any significant association between AMH concentration and embryo quality based on the scores generated by time-lapse imaging (28). Similarly, we did not find any correlation between embryo morphokinetics and AMH level, except for t5, t8, and cc3 which was negatively affected by the level of AMH. Among these variables, t5 was reported as the main parameter for prediction of implantation in ART cycles (29). 

The interpretation of these correlations is challenging due to the lack of similar studies in the literature and further studies in this field are required. Otherwise, in this study, we found a significant correlation between AMH and embryo number in both groups. Previously several reports confirmed the significant relationship between AMH and the number of embryos (7, 30) along with embryo morphological scores (31) and blastocyst formation (8). It should be considered that these studies have been performed based on traditional embryo morphological assessment which is limited to its natural inter and intra-observer variability. However, some studies that likewise used embryo morphology evaluation could not catch any association between AMH and embryo quality (32, 33). Moreover, we found a significant correlation between AMH concentration and the number of oocytes retrieved and MII oocytes. These results are in line with previous studies which showed a significant positive correlation between AMH concentration and the number of retrieved and MII oocytes (27, 34, 35). 

The present study revealed that the AMH was fair but not a powerful predictor of clinical pregnancy and live birth in TF infertility women. Some previous studies reported that AMH is a significant predictor of clinical pregnancy (12, 13, 34) as well as a live birth (27, 36, 37). Nonetheless, other studies did not show any association between AMH level and clinical pregnancy (8, 14, 32). In agreement with our findings, two meta-analyses indicated that AMH is a weak predictor of clinical pregnancy (17) and live birth (38). Furthermore, we examined the predictive ability of AMH for clinical pregnancy and live birth in PCOS women and noted a positive albeit poor correlation between AMH and ICSI outcomes. In the same way, several reports displayed that AMH concentration was not a reliable predictor of IVF outcome in women with PCOS (39-41). 

In contrast, Tal and colleagues showed a correlation between AMH levels and clinical pregnancy (42). Moreover, a meta-analysis revealed a weak prediction value of AMH for clinical pregnancy among PCOS patients (17). The poor predictive capability of AMH for pregnancy in women with PCOS could be described by the adjacent relationship between AMH and the pathogenesis of the syndrome. Women with PCOS are known by increased serum AMH levels, which have been correlated to the disease severity, and to the all of its clinical diagnostic criteria including polycystic ovarian morphology, oligo/anovulation, and hyperandrogenism (17).

The novelty of the current study is the application of time-lapse imaging for assessing embryo quality. All the previous studies evaluated embryo development by conventional morphological scores. The main limitation of this study is the small sample size which may affect the power of AMH for prediction ICSI outcome. The current study may possibly be a prospective pilot study and may help to estimate the size and design of future studies.

## Conclusion

In conclusion, for the first time, this study suggests that AMH has some correlation to embryo morphokinetics in ICSI cycles among PCOS patients, but it could not be considered as a marker of embryo quality in these patients. Our study also revealed that AMH is a weak predictor of clinical pregnancy and live birth in PCOS patients.

## Note

***This article extracted from Ph.D. thesis. (Nasim Tabibnejad)***
